# Author Correction: Air-stable superparamagnetic metal nanoparticles entrapped in graphene oxide matrix

**DOI:** 10.1038/s41467-019-10702-2

**Published:** 2019-06-18

**Authors:** Jiří Tuček, Zdeněk Sofer, Daniel Bouša, Martin Pumera, Kateřina Holá, Aneta Malá, Kateřina Poláková, Markéta Havrdová, Klára Čépe, Ondřej Tomanec, Radek Zbořil

**Affiliations:** 1 Department of Experimental Physics and Department of Physical Chemistry, Regional Centre of Advanced Technologies and Materials, Faculty of Science, Palacky University in Olomouc, Slechtitelu 27, 783 71 Olomouc, Czech Republic; 2 Department of Inorganic Chemistry, University of Chemistry and Technology Prague, Technická 5, 166 28, Prague 6, Czech Republic; 3 Division of Chemistry & Biological Chemistry, School of Physical and Mathematical Sciences, Nanyang Technological University, 637371 Singapore, Singapore; 4 Institute of Scientific Instruments, The Czech Academy of Sciences, Kralovopolska 147, 612 64 Brno, Czech Republic

Correction to: *Nature Communications* 10.1038/ncomms12879, published online 15 September 2016.

The Supplementary Information associated with this Article contains an error in Supplementary Fig. 6, in which the data in panel a (‘fresh sample’) are inadvertently copied to panels b (‘stored in air’) and c (‘stored in the physiological solution’). The correct version of Supplementary Fig. 6 can be found as Supplementary Information associated with this Correction. The error has not been corrected in the Supplementary Information associated with the Article.

## Supplementary information

Supplementary Information

