# Supplementary material for: Author Correction: Air-stable superparamagnetic metal nanoparticles entrapped in graphene oxide matrix
Source: Nat Commun. 2019 Jun 18;10:2696. doi: 10.1038/s41467-019-10702-2 (PMC6581903; doi:10.1038/s41467-019-10702-2)
Supplement: Supplementary file 1 — Supplementary Information [file 41467_2019_10702_MOESM1_ESM.pdf]

Correction of Supplementary Figure 6 – ncomms12879

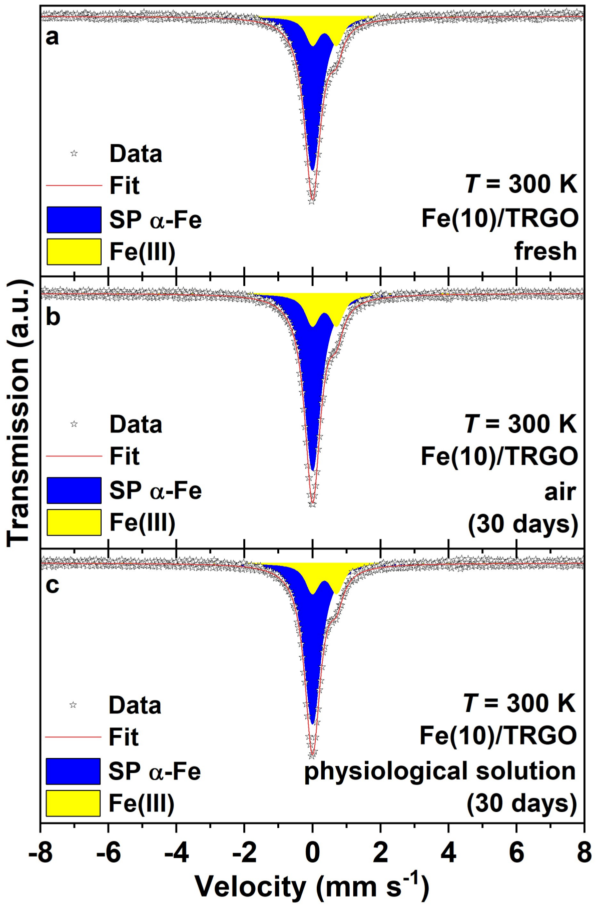

Supplementary Figure 6 | Chemical stability of the Fe(10)/TRGO hybrid under air conditions and in a physiological solution assessed by ^{57}Fe Mössbauer spectroscopy. Room-temperature ^{57}Fe Mössbauer spectra of (**a**) the freshly-prepared Fe(10)/TRGO hybrid after primary purification, (**b**) the hybrid after one month of storage under ambient laboratory conditions in the air, and (**c**) the hybrid after one month of storage in a physiological solution.
